# On misconceptions about the Brier score in binary prediction models^☆^

**DOI:** 10.1016/j.gloepi.2025.100242

**Published:** 2026-01-07

**Authors:** Linard Hoessly

**Affiliations:** Data Center of the Swiss Transplant Cohort Study, University hospital Basel, Basel, Switzerland

## Abstract

The Brier score is a widely used metric in epidemiological and clinical research for evaluating the accuracy of probabilistic predictions for binary outcomes, such as disease occurrence, treatment response, and screening performance. Despite its popularity, the Brier score is frequently misunderstood, leading to flawed interpretation of prediction models and potentially misguided public health and clinical decisions. This study aims to didactically clarify common misconceptions about realised Brier scores and to provide practical, statistically rigorous guidance for its correct interpretation in epidemiologic and public health prediction models. We analytically examined its statistical properties and conducted simulation studies across diverse scenarios, varying the distribution of true outcome probabilities, prediction accuracy, sample size, and event prevalence. Five prevalent misconceptions were identified, including the mistaken belief that a Brier score of zero indicates a perfect model. Analytic arguments and simulations demonstrated that even perfectly specified models yield non-zero Brier scores under realistic conditions. The Brier score was shown to reflect not only prediction accuracy but also the underlying distribution of true risks and random variation in outcomes. Comparisons across different populations or disease settings can therefore be misleading, and the Brier score does not directly measure calibration. We recommend restricting comparisons to the same population and complementing the Brier score with calibration metrics and measures of clinical or public health utility. Adopting these practices will improve the validity and interpretability of risk prediction in epidemiologic research and enhance decision-making in population health.

## Introduction: What is the Brier score?

The Brier score [1] is a widely used metric evaluating the accuracy of probabilistic predictions in binary outcomes for clinical research [2], [3]. It assesses the overall performance of prediction models that estimate the likelihood of medical outcomes like disease progression or treatment response.

Given probabilistic predictions $p_{i}$ and observed outcomes $y_{i}$, the Brier score is defined as:

(1) $$BS\left(p,y\right)=\frac{1}{n}\overset{n}{\underset{i=1}{\sum }}{\left(p_{i}-y_{i}\right)}^{2}.$$

where:

• n is the total number of predictions and observations,
• $p_{i}$ represents the predicted probability of an event occurring for the ith case (e.g., the probability of a patient developing a condition),
• $y_{i}$ is the actual observed outcome (coded as 1 if the event occurred, 0 if it did not).

Typically, the $y_{i}$ in such prediction models are assumed to be realisations of independent Bernoulli random variables $Y_{i}∼Bern\left(q_{i}\right)$, where $q_{i}\in \left[0,1\right]$ [3], [4]. A Bernoulli random variable is like a potentially biased coin flip: each patient either has the event 1 or 0, based on their individual risk. Two random variables are independent if the probability of an outcome for one is unaffected by the outcome of the other [5]. Correspondingly the best ith prediction that can be obtained is the true underlying probability, i.e, $p_{i}=q_{i}$.

Brier scores offer a comprehensive evaluation of probabilistic predictions and are strictly proper [6, Theorem 1], meaning that in expectation it is minimised if and only if the predictions are the true probabilities [7]. Note the distinction between accurate probabilistic predictions and clinical usefulness: while clinicians may prefer binary classifications, the quality of a probability prediction is judged by how closely its probabilities reflect the true risks. The Brier score is an evaluation measure that quantifies this closeness of predictions to true risks. If desired, predicted probabilities can be assessed for clinical impact, e.g., via net benefit [8], or used to derive a classification [9].

Despite its widespread use [10], [11], [12], the Brier score is potentially often misunderstood in clinical research. Unlike more familiar statistical notions, it does not fit neatly into traditional statistical concepts potentially commonly taught in medical education [13], [14], [15], [16]. Furthermore, the Brier score is mathematically equivalent to the mean squared error (MSE), a concept introduced by C.F. Gauss [17]. The MSE is widely applied in ordinary least squares regression [18, § 11.3.1], statistical learning [19, § 2.2.1], or machine learning evaluation [20]. The connection of Brier score and MSE can also lead to confusion. Brier score and MSE are used in different contexts, as the Brier score compares a probability to an outcome of the binary random variable in the sense of scoring rules [7], while the MSE usually compares two real continuous values, in statistics typically comparing an estimator to the true value [18]. In particular, misconceptions about the Brier score are not uncommon and can sometimes be reinforced by potentially misleading statements in the literature [2], [8], [21], [22], [23], [24]. Given the importance of accurate interpretation in clinical applications, it is crucial to address these misunderstandings.

Evaluation of binary prediction models has been widely studied in the medical literature. A review of traditional and modern performance measures is given in [8], which are also discussed in Harrells book [3, § 10] for regression models. Steyerberg’s book [2] offers a comprehensive guide to clinical model development and validation. Alternative classification metrics such as the Gini coefficient and Pietra index were examined in [25]. Other works explore alternative evaluation scores [26] and compare metrics like the Brier score with net benefit analysis [27]. The Brier score has also been decomposed for deeper insights [28], and remains relevant in AI-based medical prediction [29] or survival outcome evaluation [22]. Furthermore, fields such as meteorology and econometrics also use Brier scores or baseline-normalised measures like the Brier Skill Score for forecast verification [30]. Although conceptually related, they typically focus on single-outcome forecasts over time in different contexts, so we restrict our discussion to the medical prediction framework.

We aim to didactically clarify common misunderstandings about the Brier score for practitioners, explain why they arise, and provide guidance on its appropriate interpretation in clinical research. As it is also directed at researchers in medicine, we first introduce some notions, and give a summary of the misconceptions. Then we discuss aspects of the Brier score, give a description of the simulations before giving the misconceptions. We end with a conclusion, also giving a brief summary table of recommendations. Supplementary analysis is kept in the Supplementary information for the interested reader.

### Glossary of key terms we will use and what they mean

• **Random variable:** A random variable is a quantity that depends on the outcome of a random process. For example, let Y denote whether a leaving patient is readmitted within 30 days:
  $$Y=\left\{\begin{array}{cc} 1\; & \text{if readmitted}\\ 0\; & \text{if not readmitted} \end{array}\right)$$
  Before observation, Y is unknown and varies due to chance. If Y∼Bern(q) for q=0.2, the probability to observe a 1 is 20%, and the probability to observe a 0 is 80%.
• **Expectation:** Expectation is a way to describe the average outcome we expect over the long run, if we repeat a situation many times under the same conditions, denoted by E(⋅). The expectation of Y∼Bern(q) for q=0.2, E(Y), is 0.2.
• **Perfect prediction:** In clinical prediction models, a perfect prediction means that the predicted probabilities of outcomes exactly match the true underlying risk for each individual patient. In the case of our setting of the Brier score, if the true probabilities are given by $q=\left(q_{1},\ldots ,q_{n}\right)$, the perfect prediction is given by $p=\left(p_{1},\ldots ,p_{n}\right)=\left(q_{1},\ldots ,q_{n}\right)$. As an example, suppose a model estimates that a patient has a 20% chance of being readmitted to the hospital within 30 days. If that patient’s true risk is exactly 20%, then the model has made a perfect prediction for that individual.

### Quick reference on common misconceptions

See Table 1.

**Table 1:** Common misconceptions about the Brier score and how they contrast with statistical reality.

Misconception	Reality
# 1: Brier score of 0 = perfect model	A Brier score of 0 implies extreme predictions (0% or 100%) that exactly match outcomes. This is unusual in practice and might indicate errors.
# 2: Lower Brier score always means a better model	A lower Brier score can be misleading across datasets with different underlying distributions for the outcomes. It is only meaningful to compare Brier scores within the same population and context.
# 3: A low Brier score indicates good calibration	Calibration and Brier score measure different aspects. Calibration refers to how well predicted probabilities reflect observed risks; a model can have a low Brier score and still be poorly calibrated.
# 4: A Brier score near $\overset{¯}{y}-{\overset{¯}{y}}^{2}$ means the model is useless	Even perfect predictions can yield a Brier score near $\overset{¯}{y}-{\overset{¯}{y}}^{2}$ if the true risks are close to the mean incidence. This does not necessarily imply non-informativeness.
# 5: Brier score cannot exceed $\overset{¯}{y}-{\overset{¯}{y}}^{2}$ for reasonable predictions	As a realisation of a random variable, the Brier score can exceed the threshold due to chance or reasonable predictions.

### Related literature

Some of our points have been previously observed. We review related references that observe similar findings. However, given the widespread use of the Brier score, our literature review is necessarily partial. [31] outlines examples where the model comparison of expected Brier scores is potentially contrary to how a human would judge. [4] outlined the distinction between calibration and prediction error, emphasising the misunderstanding that low Brier score indicates good calibration, while [32] notes that Brier score comparisons across datasets should be avoided as it depends on the incidence rate. Moreover, the general point that realised outcomes can deviate substantially from their expectations is well known and has been noted repeatedly in practice; see, e.g., [33].

## Main properties of the Brier score

The Brier score in (1) is a measure to quantify the accuracy of probabilistic predictions, taking values between 0 and 1 with lower values indicating better performance. As the $y_{i}$ are realisations of random variables, the Brier score is a random variable. Hence any evaluation of the Brier score has a random component. It is strictly proper, meaning in expectations the perfect prediction uniquely minimises the Brier score of (1). We can further quantify the expected behavior when slightly perturbing the predicted probabilities away from the true values by ɛ. This results in an expected Brier score increase of ${ɛ}^{2}$. To be more specific, if, say, the predicted probabilities are 0.1 more off, the expected Brier score will be $0.1^{2}=0.01$ bigger. In contrast, changes in the true probability can lead to more significant shifts in expectation.

While individual true probabilities are unobservable in practice, the observed prevalence provides an estimate of the expected prevalence and can serve as a non-informative reference point for comparison. In idealised settings, standard large-sample arguments (via the law of large numbers and the central limit theorem [34]) can be used to argue that the observed score is a reliable estimate of its expected value. For large datasets, this can be used to justify treating the empirical Brier score as a stable summary measure of model performance. To summarise and simplify our previous points, we note that an observed Brier score is a function of

(I) the underlying true probabilities (the $q_{i}$s),
(II) the closeness of the predictions when compared to the true probabilities (how close $p_{i}$ is from $q_{i}$),
(III) some randomness that comes from the Bernoulli random variables (the observed $y_{i}$ that are realisations of $Y_{i}∼Bern\left(q_{i}\right)$).

The influence of the randomness can be expected to decreases with n, and the expectation of the Brier score can be seen as the long term average, which represent typical values if n is big. More details on properties as well as calculations for the derivations above can be found in Supplementary information together with connections to other measures.

Finally, we note that for the Brier score even more holds than strict properness: in expectation the Brier score preserves the Euclidean distance order $l_{2}$ between predictions $p\in {\left[0,1\right]}^{n}$ to the true probability $q\in {\left[0,1\right]}^{n}$, where $l_{2}\left(p,q\right)=\sqrt{\sum_{i=1}^{n}{\left(p_{i}-q_{i}\right)}^{2}}$ [35].

Suppose we have two sets of predicted probabilities, p and $p^{\prime }$, from two different models, and let q represent the true probabilities. If p is closer to q than $p^{\prime }$ in terms of Euclidean distance, i.e.,

$$l_{2}\left(p,q\right)<l_{2}\left(p^{\prime },q\right),$$

then the expected Brier score for p will be lower than that of $p^{\prime }$. However, note that this property is in expectation, and does not imply that the same holds for a realised Brier score.

To build intuition, consider a 2-dimensional example where the true probabilities are q=(1/2,1/2). In this case, any prediction falling inside a circle centered at (1/2,1/2) will have a lower expected Brier score than one on or outside the circle. This provides a geometric view of probabilistic accuracy: the closer your predictions are to the truth (in Euclidean distance), the better the model performs (in expectation).

**Figure:**
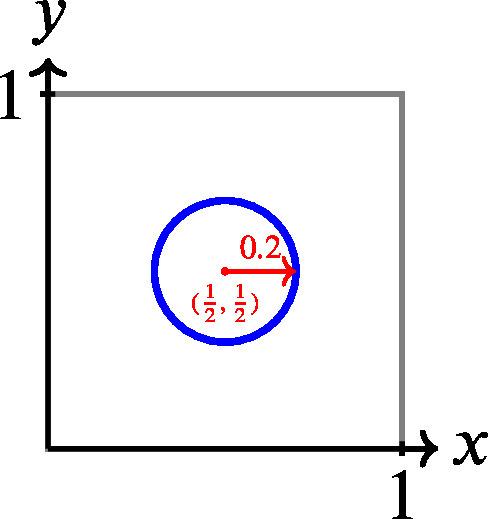

### Observed versus expected properties of the Brier score

Many properties of the Brier score, including strict properness or Euclidean distance preservation, hold in expectation under the data-generating process. However, practitioners assess realised Brier scores from finite samples, which are random and subject to sampling variability. Hence we distinguish the following aspects

#### (i) Theoretical qualities.

• In expectation, the Brier score is uniquely minimised when $p_{i}=q_{i}$.
• Expected scores preserve the Euclidean distance ordering between models.

#### (ii) Realised Brier scores.

• Observed Brier scores fluctuate due to randomness in $Y_{i}∼Bern\left(q_{i}\right)$ and may occasionally favor an inferior model.
• While standard large-sample arguments (through the law of large numbers in an idealised setting [34]) ensures convergence of the Brier score (1) to its expectation as n increases, variability can remain substantial in small samples or due to nonstandard data-generating processes.
• For realised scores the data generating process for $p_{i},q_{i}$ is unknown, and one has to quantify uncertainty in Brier scores, e.g., via bootstrap confidence intervals [2]. If the data generating process for both $p_{i},q_{i}$ is known, one can use standard large-sample arguments (through the central limit theorem [34]) to quantify uncertainty.

### Means of understanding the Brier score

We will use the following approaches to better understand the Brier score (1):

• **Expectation of the Brier score:** We will analyze the expected value the Brier score (1) takes, which will help to illustrate typical observed values of the Brier score.
• **Simulation-Based Evaluations:** We assess and illustrate the behavior of the Brier score by simulating Bernoulli outcomes under various probability distributions for $q_{i}$ and different sample sizes. A concise ADEMP-style summary of the simulation framework is provided below, while detailed specifications on setting and code are included in the Supplementary Information to maintain readability for the applied audience [36]. **A (Aims):** To illustrate and examine how the Brier score behaves across different data-generating settings and prediction perturbations. **D (Data-generating mechanism):** For each scenario, true probabilities $q_{i}\in \left[0,1\right]$ are sampled from several distributions (uniform, beta, or model-based from NHANES). Outcomes are generated as $Y_{i}∼\mathrm{Bern}\left(q_{i}\right)$. Predicted probabilities $p_{i}$ are defined as functions of $q_{i}$ with various perturbations (additive bias, random noise, or symmetric logit-scale error). Sample sizes n∈{300,1000} are considered, with 5000 Monte Carlo replicates per scenario. **E (Estimands):** The main estimands are the distribution, median, and central 90% interval of the observed Brier score. **M (Methods):** Simulations were implemented in R [37]. For NHANES-based scenarios, true probabilities $q_{i}$ were obtained from fitted logistic regression models and subsampled without replacement; for other scenarios, $q_{i}$ were drawn i.i.d. from the specified distributions. **P (Performance measures):** The empirical distributions of observed Brier scores are summarised using violin plots, medians, and the 5th and 95th percentiles. Full implementation details and code are provided in the Supplementary Information.

## Misconceptions

Below are the most common misinterpretations of the Brier score when evaluating probability predictions for binary events, accompanied by an explanation and examples illustrating why it is incorrect.

### Misconception #1: A Brier score of 0 means a perfect model, and a perfect model has Brier score 0

• **A Brier score of 0 means a perfect model.** A Brier score of 0 implies perfect alignment between predicted probabilities and observed outcomes, with predictions exclusively 0 or 1. The true probabilities are within [0,1], usually expected in (0,1). Hence, rather than signaling a perfect model, an observed Brier score of 0 should raise strong suspicion of model misspecification or numerical errors. However, although classification settings were outside our scope, such cases cannot be fully excluded in practice.
• **A perfect model has Brier score 0.** With at least one of the true probabilities $q_{i}$ in (0,1), observing a Brier score of 0 with perfect predictions is impossible (see Supplementary information). Hence in normal situations perfect models will have a Brier score bigger than 0.

To illustrate this and for later reference, we present simulations with perfect predictions, showing that ideal models do not yield a Brier score of 0. In fact, expected Brier scores for a perfect model can be notably high.

**Fig. 1:**
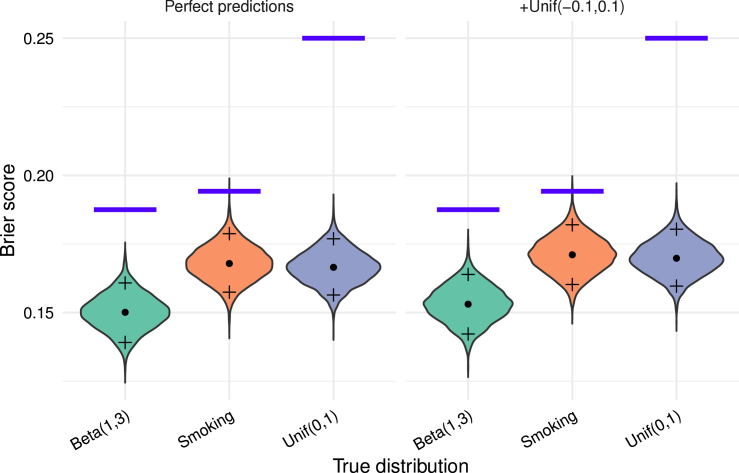
Visualisation of the distribution of observed Brier score through a violin plot for simulations with n=1000 across 5000 Monte-Carlo replicates. The dot corresponds to the median and the crosses above and below to 95% percentile resp. 5% percentile. The left side has perfect predictions, the right Unif(−0.1,0.1) noise added. The blue bar corresponds to the theoretical value of the upper limit of the expected Brier $\overset{¯}{q}-{\overset{¯}{q}}^{2}$ of the true distribution.

### Misconception #2: When comparing two prediction models, the model with lower Brier score is better

Brier scores enable model comparison across datasets, but comparisons can be misleading. We examine three scenarios of increasing risk to illustrate this point.

#### Comparing models on the same dataset via Brier score

When evaluating two models on the same dataset, lower Brier score indicate better fit. There are two potential issues that can prevent the better model to have lower Brier score:

(I) It is possible that the worse model has a better score by chance. However, with higher sample sizes this becomes more unlikely.
(II) In expectation the Brier score ranks according to the $l_{2}$-distance between prediction $p\in {\left[0,1\right]}^{n}$ to the true probability $q\in {\left[0,1\right]}^{n}$. Changing perfect predictions from $q_{i}$ to $q_{i}+ɛ$ or $q_{i}-ɛ$ gives the same result in expectation. However, for humans the direction can matter particularly for $q_{i}$ close to zero or one [31]. Consider the example with one observation from [31, § 3] where the probability of an event is $P\left(Y_{1}=1\right)=\frac{1}{10}$ (i.e., 10% chance), and compare two models:
  • Model 1: $p_{1}=0$ (predicting the event will never occur)
  • Model 2: $p_{1}^{\prime }=\frac{1}{4}$ (predicting the event occurs with a probability of 25%)
  Model 1 has an expected Brier score of 0.1, as compared to 0.1125 for model 2. However, model 1 predicts a zero probability for the observation that has actually a 10% probability, hence 25% might seem better from a humans perspective.

#### Comparing Brier scores across different datasets with similar incidences (also known as class imbalance) is meaningful

Some recommend to compare Brier score of prediction models on datasets with similar class imbalances [24]. However, the true probability distribution is unobservable, and true values $q_{i}$ strongly influences the expectation of the Brier score. Thus, even with identical class imbalances, we cannot assume that the true probability distributions are comparable and the same caution as in the previous point should be exercised in interpreting such comparison. As an example, compare three cases: True distribution always 1/2, independent Unif(0,1) from Fig. 1, or a 0 or 1 distribution with each 50%. The perfect predictions have in each case expected Brier score 0.25,0.16 and 0. Hence perfect predictions for true distribution always 1/2 and expected Brier score 0.25 is better than, say predictions with noise Unif(−0.1,0.1) for true probabilities Unif(0,1) from Fig. 1 with expected Brier score 0.17. Potentially, similarities in outcome and population trough population characteristics might indicate how true probabilities differ or align in a clinical prediction model setting.

#### Comparing Brier scores across datasets with different incidences

If true outcome distributions differ, even perfect models yield different Brier score distributions and (potentially) expectations. Thus, cross-dataset comparisons may lack meaningful insight. Observing different incidences may indicate that the true outcome distributions differ, making the Brier score comparison unreliable. Compare, e.g. the Beta(1,3) prediction with prediction error +Unif(−0.1,0.1) to the perfect smoke prediction model from Fig. 1. The Beta(1,3) prediction with prediction error has lower Brier score mostly when compared to perfect smoking prediction, nonetheless the perfect model is obviously better.

### Misconception #3: A low Brier score indicates good calibration

**Fig. 2:**
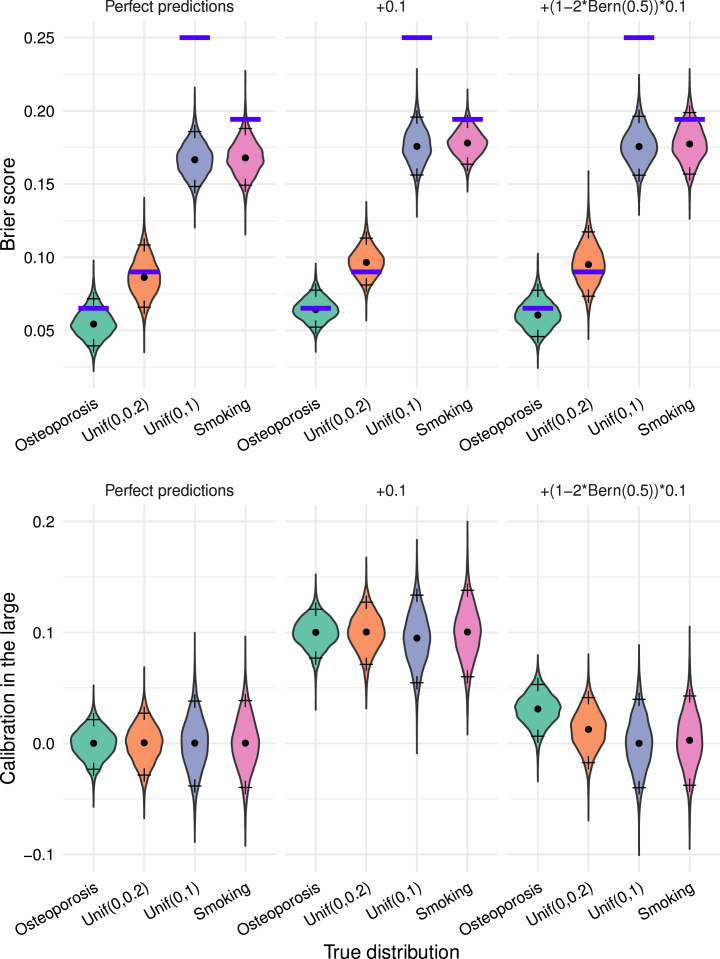
Visualisation of the distribution of Brier scores above and CIL below through violin plots for simulations with n=300 across 5000 Monte-Carlo replicates. The dot corresponds to the median and the crosses at above and below to 95% percentile resp. the 5% percentile. The blue bar above corresponds to the theoretical value of the upper limit of the expected Brier $\overset{¯}{q}-{\overset{¯}{q}}^{2}$ of the true distribution.

A low Brier score does not necessarily indicate good calibration of a model. We can have perfect predictions, but low or high Brier score due to the underlying probabilities, or similarly not so accurate or biased predictions but a Brier score that is low or high due to the underlying probabilities. In expectation a change of perfect prediction from $p_{i}=q_{i}$ to $q_{i}+ɛ$ or $q_{i}-ɛ$ gives the same result (see Supplementary information), and miscalibration where errors tend to go mostly in one direction are equally punished, but e.g. calibration is differently affected as illustrated in Fig. 2.

Assessing calibration should be done using additional metrics like calibration in the large (CIL), calibration curves or similar evaluation components [38], [39]. Hence Brier score should not be the sole criterion for evaluating model performance.

Example 1 We give an illustrative example with N=2, and once perfect and once biased calibration where the biased case has lower expected Brier score. Consider two settings with different underlying risks.

• *Setting A*: True and predicted probabilities coincide with q=p=(0.3,0.7), yielding an expected Brier score of 0.21 with an expected CIL of zero.
• *Setting B*: True probabilities are $q^{\prime }=\left(0.2,0.8\right)$ but predictions $p^{\prime }=\left(0.444949,1\right)$, which systematically overestimates risk. This gives an expected Brier score of 0.2 but expected CIL of 0.2224745.

Despite a higher expected Brier score in setting A, its predictions have perfect calibration, while in setting B the predictions are systematically overestimating. This illustrates that low Brier scores can conceal systematic bias in predicted probabilities.

Finally, these observations relate to Misconception #1, addressing the mistaken belief that a Brier score of zero signifies a perfect model.

### Misconception #4: Having a Brier score of around $\overset{¯}{y}-{\overset{¯}{y}}^{2}$ where $\overset{¯}{y}$ is the mean observed incidence means we have a useless or non-informative model. As an example, if $\overset{¯}{y}=0.5$, then $\overset{¯}{y}-{\overset{¯}{y}}^{2}=0.25$, or, if $\overset{¯}{y}=0.1$, then $\overset{¯}{y}-{\overset{¯}{y}}^{2}=0.090$

Note that the lowest expected Brier score occurs when predicted probabilities match the true probabilities, representing a perfect prediction that cannot be improved. However, we cannot observe the true probabilities. With observed incidences of 0.5, perfect predictions can yield a Brier score of close to 0.25. Hence if we observe a Brier score of around $\overset{¯}{y}-{\overset{¯}{y}}^{2}$, the following alternatives to a bad model could explain such a Brier score:

• Many of the true probabilities are around $\overset{¯}{y}$, making expected Brier scores of perfect predictions close to $\overset{¯}{y}-{\overset{¯}{y}}^{2}$.
• For n low, randomness can make the Brier score higher than its expectation.

**Fig. 3:**
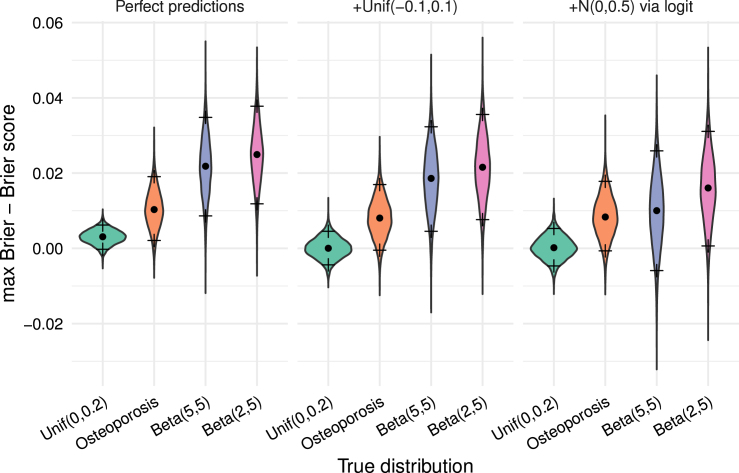
Visualisation of the distribution of $\overset{¯}{y}-{\overset{¯}{y}}^{2}-BS_{perf}$ through a violin plot for simulations with n=300 across 5000 Monte-Carlo replicates. The dot corresponds to the median and the crosses above and below to 95% percentile resp. the 5% percentile.

As an illustration of example values, consider $\overset{¯}{y}-{\overset{¯}{y}}^{2}-BS_{perf}$ in Fig. 3 on the left, where $BS_{perf}$ is the Brier score under perfect predictions. The observed median is very low, with 5% percentile below or around zero.

### Misconception #5: For an observed incidence of $\overset{¯}{y}$, the Brier score (1) for reasonable predictions cannot be bigger than $\overset{¯}{y}-{\overset{¯}{y}}^{2}$

**Fig. 4:**
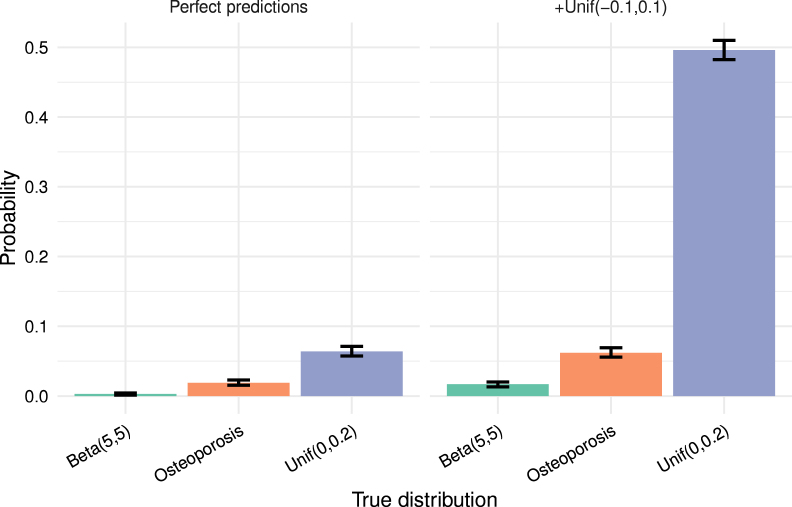
Histogram for empirical probability of the events $\overset{¯}{y}-{\overset{¯}{y}}^{2}>BS$ for simulations with n=300 across 5000 Monte-Carlo replicates. The bars indicate 95% bootstrap confidence intervals.

Although the observed Brier score might often be bounded above by $\overset{¯}{y}-{\overset{¯}{y}}^{2}$ in practice, higher values can occur. Since true probabilities $q_{i}$ are unobservable, we cannot rule out that even perfect predictions yield a score near the unobservable $\overset{¯}{q}-{\overset{¯}{q}}^{2}$, where $\overset{¯}{q}$ is the true mean incidence $\overset{¯}{q}=\frac{1}{n}\sum_{i=1}^{n}q_{i}$. The Brier score’s randomness stems from the outcomes, also making $\overset{¯}{y}$ random, and making $\overset{¯}{y}-{\overset{¯}{y}}^{2}$ a noisy estimate of $\overset{¯}{q}-{\overset{¯}{q}}^{2}$. Hence a Brier score exceeding $\overset{¯}{y}-{\overset{¯}{y}}^{2}$ may also result from the same options as given in misconception #4. We illustrate this in Fig. 4: even with perfect predictions in some settings with n=300, the probability that the Brier score exceeds $\overset{¯}{y}-{\overset{¯}{y}}^{2}$ is nonzero. In practice, predictions will not be perfect; with reasonably accurate (but imperfect) predictions, the probability that the Brier score exceeds $\overset{¯}{y}-{\overset{¯}{y}}^{2}$ will typically be larger. The right part of Fig. 4 can therefore be interpreted as providing potential lower bounds for these probabilities in the respective settings.

## Conclusions and final remarks

We addressed common misconceptions regarding the interpretation and use of the Brier score. The Brier score is a realisation of a random variable. The true underlying probabilities influence the expectation of Brier score strongly, potentially stronger than the closeness of the predictions to the true probabilities. Brier scores of zero can be an indication of errors in realistic settings, and a low Brier score does not necessarily indicate a perfect model. Comparisons of Brier scores across models on the same data can be done, and across different data should be avoided or interpreted carefully. Brier scores may be compared to the score based on the observed prevalence, be it directly or through the scaled Brier score [10]. Note that although related to the Brier Skill Score, the scaled Brier is mathematically distinct [40]. Finally, cross-dataset comparison using the scaled Brier, are not recommended as the expected Brier score of perfect predictions depends on the underlying distribution. Low Brier scores do not guarantee good calibration as evaluating calibration should be done with other metrics. In analogy to idealised settings, randomness in observed Brier scores can be expected to decrease with bigger sample sizes. A recent literature review on clinical prediction models found that sample sizes used had median sample size of 1250 with (Q1,Q3)=(353,188860) [41]. Hence at least a quarter of the prediction models had relatively low sample sizes, with randomness similar to the settings in our simulations with n=300 or n=1000. Hence, Misconceptions #4 and #5 are likely rarer in clinical prediction models, as they tend to arise mainly in smaller samples.

The Brier score remains a valuable metric for assessing probabilistic predictions, but its interpretation requires an understanding of how the underlying probabilities and closeness of predictions influence the observed value. Once misconceptions are avoided, the Brier score serves as a reliable relative measure of overall performance. In particular, it is effective and strictly proper and hence in expectation, it reflects Euclidean distance between predictions, and is minimised uniquely at the true probabilities making relative comparisons on the same data meaningful.

### Recommendations for applied use

See Table 2.

## Declaration of competing interest

The authors declare that they have no known competing financial interests or personal relationships that could have appeared to influence the work reported in this paper.

**Table 2:** Recommended best practices for interpreting and reporting the Brier score in applied research.

Recommendation	Rationale/Comment
# 1: Benchmark Brier scores within the same population and against a prevalence-based baseline.	Compare model Brier scores on the same data, potentially also to a simple baseline predicting constant prevalence. This ensures the score reflects improvements in prediction with the Brier score.
# 2: Avoid cross-population comparisons	The Brier score depends on the underlying outcome distribution; comparing and ranking models across populations or prevalences can be misleading.
# 3: Combine with other evaluation measures	When evaluating predictions through Brier, we project to one dimension. Hence it is good to combine the Brier score with other measures, e.g., calibration using CIL or calibration plots, discrimination using c-index, and, e.g., net benefit or decision curve analysis for clinical utility to get a more comprehensive understanding of the predictions [2].
# 4: Quantify uncertainty in observed Brier scores	Report bootstrap confidence intervals to reflect sampling variability.

## Supplementary data

MMC S1 Supplementary Information: Brier score properties, expectations, related metrics, proofs, and simulation details (ADEMP) MMC S1
