# Supplementary material for: On misconceptions about the Brier score in binary prediction models
Source: Glob Epidemiol. 2026 Jan 7;11:100242. doi: 10.1016/j.gloepi.2025.100242 (PMC12818272; doi:10.1016/j.gloepi.2025.100242)
Supplement: MMC S1 — Supplementary Information: Brier score properties, expectations, related metrics, proofs, and simulation details (ADEMP) [file mmc1.pdf]

SUPPLEMENTARY INFORMATION

Appendix A. More on the Brier score

In this section, we review key properties of the Brier score, delve into some mathematical points, and give more details on the simulations we use to visualise the behavior of the Brier score.

$$BS(p, y) = \frac{1}{n}\sum_{i=1}^{n}(p_i - y_i)^2. \tag{A.1}$$

When evaluating the Brier score (A.1), one can compare (A.1) to the trivial Brier score when entering $p_{1/2} = (1/2, \cdots, 1/2)$, which, independent of the specific value of y gives $BS(p_{1/2}, y) = 1/4$. A slightly better prediction is the mean incidence $\bar{y}_v = (\bar{y}, \cdots, \bar{y})$, which when used in the Brier score gives

$$BS(\bar{y}_v, y) = \bar{y} - \bar{y}^2.$$

Clearly, $BS(p_{1/2}, y) = 1/4 \geq BS(\bar{y}_v, y) = \bar{y} - \bar{y}^2$. For high or low incidences, $BS(\bar{y}_v, y)$ is low, and, e.g., for $\bar{y} = 0.1$ or $\bar{y} = 0.9$, $BS(\bar{y}_v, y) = 0.09$. Furthermore, $\bar{y} - \bar{y}^2$ is symmetric around 0.5, see Figure 1.

A.1. **Key properties about the Brier score.** We summarise essential properties of the Brier score.

- **(BI) Range and interpretation:** The Brier score takes values in the interval $[0, 1]$, with lower values typically indicating more accurate probabilistic predictions.
- **(BII) The Brier score is a random variable:** As mentioned in main text, the Brier score is a function of random variables and hence a random variable
- **(BIII) Optimal predictions and true probabilities:** As mentioned in main text, the unique optimal prediction minimising the expected Brier score is the true outcome probability, i.e., for all i $p_i = q_i$ [4, Theorem 1].
- **(BIV) Expectation of Brier score:**
  - ($n = 1$): As an illustrative example consider the case $n = 1$. Using the true probability, we calculate the expectation. Let $Y_1 \sim Bern(q_1), q_1 \in [0, 1]$ with prediction $p_1 \in [0, 1]$. Then the expectation of Brier score is

$$g(p_1, q_1) := \mathbb{E}_{Y_1 \sim Bern(q_1)}[BS(p_1, Y_1)] = p_1^2 - 2p_1 q_1 + q_1. \tag{A.2}$$

    For the interested reader, the calculation is given in Appendix D.1. The optimal prediction minimising (A.2) is $p_1 = q_1$. We next go through two key cases
    - For perfect prediction, the expectation value is given by

$$f(q_1) = q_1 - q_1^2. \tag{A.3}$$

      As examples, consider the following cases:
      * if $q_1 = 1/2 = p_1$, the expected Brier score is $f(1/2) = 1/4$ corresponding to the maximum of (A.3),
      * if for $q_1 = 1/10 = p_1$, $f(1/10) = 9/100$.

      The expectation of the Brier score as a function of q_1, when $p_1 = q_1$, i.e., (A.3), looks as follows:

    - Next we compare the expected Brier score under perfect prediction with $p_1 = q_1$ to
      * the expectation of the Brier score under perfect prediction but increased true probability, i.e., $\tilde{p}_1 := p_1 + \varepsilon = \tilde{q}_1 = q_1 + \varepsilon$ for $q_1 + \varepsilon \leq 1/2$, and taking the difference

$$g(q_1 + \varepsilon, q_1 + \varepsilon) - g(q_1, q_1) = \varepsilon(1 - 2q_1 - \varepsilon) \tag{A.4}$$

        Note that the difference depends strongly on q_1, and that the same holds if we shift true probability and perfect prediction from $q_1 > 1/2$ to some value $q_1 - \varepsilon$ for $q_1 - \varepsilon \geq 1/2$.

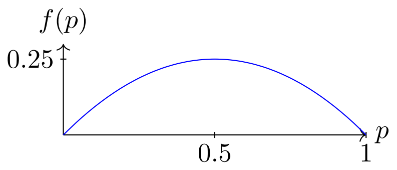

FIGURE 1. Expectation of the Brier score for the optimal prediction as a function of the underlying true probability with $p_1 = q_1$.

∗ to the expectation of the Brier score with same true probability but slightly wrong prediction $\tilde{p}_1 := p_1 + \varepsilon, \tilde{q}_1 = q_1 \in [0,1]$ and taking the difference

$$g(q_1 + \varepsilon, q_1) - g(q_1, q_1) = \varepsilon^2 \tag{A.5}$$

Note that the difference does not depend on q_1, and the same holds for $\tilde{p}_1 := p_1 - \varepsilon$. The calculations are in Appendix § D.2.

– From the previous point, we conclude that if we slightly predict wrong, the costs are almost inexistent, e.g. if the true probability is q_1 but we predicted $p_1 = q_1 + 0.1$, the expected difference between the Brier score of the perfect prediction and the slightly wrong one is 0.01 by (A.5). However, if the true probability is changed towards 0.5, e.g. from $q_1 = 0.1$ to $\tilde{q}_1 = 0.2$ and we have perfect prediction, the expected difference of the Brier scores is 0.07 by (A.4) which is seven times more.

- n arbitrary: As the Brier score is a rescaled sum of the Brier score with $n = 1$, the above conclusion extends in a straightforward way. We note the following nice order preservation property from (A.5): If $p_1, \tilde{p_1} \in [0,1]$ such that $|p_1 - q_1| < |\tilde{p_1} - q_1|$, then

$$\mathbb{E}_{Y_1 \sim Bern(q_1)}[BS(p_1, Y_1)] < \mathbb{E}_{Y_1 \sim Bern(q_1)}[BS(\tilde{p_1}, Y_1)].$$

This property is known as being effective [7], and for $n > 1$ the order that is preserved is the order implied by the l_2 distance from $q \in [0,1]^n$ to the prediction $p \in [0,1]^n$.

(BV) Dependence of expectation and distribution of Brier score on true probabilities: The Brier score for multiple observations, as defined in (A.1), is the mean of the individual (one-dimensional) Brier scores. Its expectation and distribution depend on the true outcome probabilities q_i, which are generally unknown.

As illustrated by (A.3) and Figure 1, the expected Brier score under perfect predictions varies with the distribution of the q_i. For example, if the q_i are mostly concentrated near 0 or 1, the expected Brier score is close to 0. In contrast, if the q_i cluster around 0.5, the expected Brier score under perfect predictions approaches 0.25.

(BVI) Unobservability of true probabilities in clinical data: In practice, the true probability of an event occurring for an individual patient is not observable. Each patient is unique, and we only observe whether the event occurs or not (i.e., a binary outcome). This is in contrast to simulation settings where one can compare the true probabilities to estimated probabilities of regression or ML models, i.e., as done here [13, 5].

While individual-level probabilities will remain unknown, we can approximate their average value across a population by calculating the mean of observed outcomes overall, or in similar patient groups. This can be used to assess model calibration in clinical settings via calibration in the large (CIL) [3, 12]

$$CIL(p, y) = \frac{1}{n}\sum_{i=1}^{n} p_i - \frac{1}{n}\sum_{i=1}^{n} y_i. \tag{A.6}$$

A.2. **Notable mathematical features about the Brier score.** Below, we summarise basic mathematical properties of the Brier score for the understanding of the reader. Mathematical details and arguments are given in Appendix D.3.

Consider the Brier score of n outcomes, where each $Y_i \sim Bern(q_i)$. Let the expected incidence be denoted by $\bar{q}$, i.e. $\bar{q} := \frac{\sum_{i=1}^{n} q_i}{n}$.

(MI) Brier score of non-informative model that uses prevalence as prediction, i.e. $p = (\bar{y}, \cdots, \bar{y})$**:** The Brier score of (A.1) with the non-informative mean as predictor $\bar{y}_v = (\bar{y}, \cdots, \bar{y})$, is given by $\bar{y} - \bar{y}^2$, i.e.

$$BS(\bar{y}_v, y) = \bar{y} - \bar{y}^2.$$

(MII) Bound on expectation of perfect prediction, i.e. for $p = (q_1, \cdots, q_n)$**:** The expected value of the average Brier score of (A.1) with the true probabilities as predictors, i.e., $p = (q_1, \cdots, q_n)$, is bounded above by $\bar{q} - \bar{q}^2$, i.e.,

$$\mathbb{E}[BS((q_1, \cdots, q_n), (Y_1, \cdots, Y_n))] \leq \bar{q} - \bar{q}^2,$$

with equality if and only if $q_i = \bar{q}$ for all $i \in [n]$.

(MIII) Typical Brier score with perfect prediction for large n**:** Assume the true probabilities q_i itself are realisations of random variables $Q_i \sim F$. Then, by the law of large numbers(LLN) the Brier score (A.1) for n big roughly equals $\mathbb{E}[BS(Q_1, Y_1)]$, and probabilities for deviation from this value can be calculated via the central limit theorem (CLT). Hence $\mathbb{E}[BS(Q_1, Y_1)]$ roughly equals $BS((q_1, \cdots, q_n), (y_1, \cdots, y_n))]$ for n large, and $\bar{y}$ roughly equals $\bar{q}$. Similarly if the predictions are given by Q_1 plus some iid error, the LLN and CLT apply, and, e.g., tails can be analysed via large deviations theory. More detail on this perspective is in Appendix § B.

Appendix B. Why we care about the expectation: law of large numbers and central limit theorem

Another way to understand the behavior of the Brier score for perfect predictions is through basic mathematical tools. Consider the setting of the simulations considered, where the true probability q_i itself is a realisation of a random variable $Q_i \sim F$. Denote by $J_n = (Q_1, \cdots, Q_n)$ the random vector of the first n true probabilities and $Z_n = (Y_1, \cdots, Y_n)$ the realisation of the corresponding n Bernoulli random variables. We can consider the Brier score of the optimal predictor J_n as

$$BS(J_n, Z_n) \xrightarrow{n \to \infty} \mathbb{E}[(Q_1 - Y_1)^2]$$

For sufficiently large n, the observed Brier score provides a stable estimate of its expectation by the LLN [8].

Furthermore, the individual terms $(Q_i - Y_i)^2$ have finite variance and hence by the CLT the distribution of the Brier score, when properly normalised, approaches a normal distribution.

$$\sqrt{n}\left(BS(J_n, Z_n) - \mathbb{E}[(Q_1 - Y_1)^2]\right) \xrightarrow{d} \mathcal{N}(0, \sigma^2), \tag{B.1}$$

where σ^2 represents the variance of $(Q_1 - Y_1)^2$, and $\xrightarrow{d}$ indicated convergence in distribution [8]. This asymptotic normality allows for understanding the asymptotic behaviour of perfect predictions in the context of the simulations.

Appendix C. Connections to some other scores

We mention three other scores connected to Brier score. The Brier score from (A.1) equals mathematically the MSE, hence its square root is the root mean squared error (RMSE):

$$RMSE(p, y) := \sqrt{BS(p, y)}. \tag{C.1}$$

As the square root on $[0, 1]$ is order preserving, i.e., if $a, b \in [0, 1], a \leq b$ then $\sqrt{a} \leq \sqrt{b}$, and bijective, RMSE also takes values in $[0, 1]$ and most of the observations we made apply similarly to RMSE.

Two similar and often-used scores are the mean-absolute error (MAE) that is defined as

$$MAE(p, y) = \frac{1}{n} \sum_{i=1}^{n} |p_i - y_i|, \tag{C.2}$$

and the CIL [11]

$$CIL(p, y) = \frac{1}{n} \sum_{i=1}^{n} p_i - y_i. \tag{C.3}$$

These relate to Brier score (or RMSE) through the following inequalities [2]

$$CIL(p, y) \leq MAE(p, y) \leq RMSE(p, y) \tag{C.4}$$

as well as

$$MSE(p, y) \leq MAE(p, y) \leq RMSE(p, y). \tag{C.5}$$

In (C.5) the first inequality holds as for $a \in [0, 1]$, we have $a^2 \leq a$, and the second inequality holds by the arithmetic mean-quadratic mean inequality that can be derived via Cauchy-Schwartz [2].

Appendix D. More detail for mathematical understanding Brier score

D.1. Expectation of Brier score in one dimension. Consider $Y_1 \sim Bern(q_1), q_1 \in [0, 1]$ and one prediction $p_1 \in [0, 1]$, then the expected Brier score is given as

$$\mathbb{E}[BS(p_1, Y)] = p_1^2 - 2p_1q_1 + q_1.$$

In order to derive the above formula, we can proceed as follows.The expected Brier score is

$$\mathbb{E}[BS(p_1, Y_1)] = \mathbb{E}[(p_1 - Y_1)^2].$$

We can expand the terms to get

$$\mathbb{E}[(p_1 - Y_1)^2] = \mathbb{E}[p_1^2 - 2p_1Y_1 + Y_1^2].$$

Since p_1, p_1^2 are constants, we get:

$$\mathbb{E}[p_1^2 - 2p_1Y_1 + Y_1^2] = p_1^2 - 2p_1\mathbb{E}[Y_1] + \mathbb{E}[Y_1^2].$$

For a Bernoulli-distributed variable Y_1:

$$\mathbb{E}[Y_1] = q_1, \quad \mathbb{E}[Y_1^2] = \mathbb{E}[Y_1] = q_1.$$

Hence the expected Brier score is:

$$\mathbb{E}[BS(p_1, Y_1)] = p_1^2 - 2p_1q_1 + q_1.$$

D.2. Differences in expectation of Brier score in one dimension. Recall that

$$g(p_1, q_1) = p_1^2 - 2p_1q_1 + q_1.$$

- To derive the first result, we compute:

  $$g(q_1 + \varepsilon, q_1 + \varepsilon) - g(q_1, q_1) = q_1^2 + 2q_1\varepsilon + \varepsilon^2 - 2(q_1^2 + 2q_1\varepsilon + \varepsilon^2) + q_1 + \varepsilon - q_1 + q_1^2$$

  Thus,

  $$g(q_1, q_1) - g(q_1 + \varepsilon, q_1 + \varepsilon) = \varepsilon(1 - 2q_1 - \varepsilon)$$

- For the second expression,

  $$g(q_1 + \varepsilon, q_1) - g(q_1, q_1) = (q_1 + \varepsilon)^2 - 2(q_1 + \varepsilon)q_1 + q_1 - (q_1^2 - 2q_1q_1 + q_1)$$

  $$= q_1^2 + 2q_1\varepsilon + \varepsilon^2 - 2q_1^2 - 2q_1\varepsilon + q_1 - q_1^2 + 2q_1^2 - q_1$$

  Thus, the difference is:

  $$g(q_1 + \varepsilon, q_1) - g(q_1, q_1) = \varepsilon^2$$

D.3. **Understanding the expectation of the general Brier score.** In case $p_1 = q_1$, the expectation value of Brier score is given by

$$f(p_1) = p_1 - p_1^2. \tag{D.1}$$

This is a strictly concave function, meaning that for any $\alpha \in [0,1]$ and any $x, y \in [0,1]$,

$$f((1-\alpha)x + \alpha y) \geq (1-\alpha)f(x) + \alpha f(y) \tag{D.2}$$

Now we come back to the case where we have n observations and we consider the Brier score.

Lemma D.1. *Consider the Brier score of n outcomes, where $\frac{\sum_{i=1}^n y_i}{n} = \bar{y}$. Then the Brier score of* (A.1) *with prevalence as predictors, i.e., $(p_1, \cdots, p_n) = (\bar{y}, \cdots, \bar{y})$ equals $\bar{y} - \bar{y}^2$, i.e.*

$$BS((\bar{y}, \cdots, \bar{y}), (y_1, \cdots, y_n)) = \bar{y} - \bar{y}^2$$

Proof. Let $n = a + b$ such that $\bar{y} = \frac{a}{a+b}$, and Brier score is given as

$$\frac{1}{a+b}(a(\frac{a}{a+b} - 1)^2 + b(\frac{a}{a+b})^2)$$

which we rewrite as

$$\frac{a}{a+b}(\frac{a}{a+b} - 1)^2 - (\frac{a}{a+b} - 1)(\frac{a}{a+b})^2) = \bar{y}(\bar{y} - 1)^2 + (1-\bar{y})\bar{y}^2 = \bar{y} - \bar{y}^2$$

□

Lemma D.2. *Consider the Brier score of n outcomes, where $\frac{\sum_{i=1}^n q_i}{n} = \bar{q}$. Then the expected value of the average Brier score of* (A.1) *with the true probabilities as predictors, i.e., $(p_1, \cdots, p_n) = (q_1, \cdots, q_n)$, is bounded above by $\bar{q} - \bar{q}^2$, i.e.*

$$\mathbb{E}[BS((q_1, \cdots, q_n), (Y_1, \cdots, Y_n))] \leq \bar{q} - \bar{q}^2,$$

with equality if and only if $q_i = \bar{q}$ for all $i \in [n]$.

Proof. Let $n \geq 1$. Then

$$\mathbb{E}[BS((q_1, \cdots, q_n), (Y_1, \cdots, Y_n))] = \frac{1}{n}(\sum_{i=1}^n q_i - q_i^2).$$

We can rewrite this as

$$= \frac{1}{n}\sum_{i=1}^n q_i - \frac{1}{n}\sum_{i=1}^n q_i^2 = \bar{q} - \frac{1}{n}\sum_{i=1}^n q_i^2.$$

As the function $x \to x^2$ is strictly convex, we can apply Jensens Inequality to get

$$\frac{1}{n}\sum_{i=1}^n q_i^2 \geq (\frac{1}{n}\sum_{i=1}^n q_i)^2 = \bar{q}^2,$$

such that finally we can bound it as

$$\bar{q} - \frac{1}{n}\sum_{i=1}^n q_i^2 \geq \bar{q} - \bar{q}^2.$$

The statement with equality if and only if $q_i = \bar{q}$ for all $i \in [n]$ also follows from Jensen. □

Another simple observation, with proof here is the following

Lemma D.3. *Consider the Brier score of n outcomes, where $\frac{\sum_{i=1}^n q_i}{n} = c$. Then the expected value of the average Brier score of* (A.1) *with the non-informative mean as predictors, i.e., $(p_1, \cdots, p_n) = (c, \cdots, c)$, is given by $c - c^2$, i.e.*

$$\mathbb{E}[BS((c, \cdots, c), (Y_1, \cdots, Y_n))] = c - c^2$$

Proof. Using (A.2) and (A.1) we get

$$\mathbb{E}[BS((c,\cdots,c),(Y_1,\cdots,Y_n))] = \frac{1}{n}\sum_{i=1}^{n} c^2 - \frac{1}{n}\sum_{i=1}^{n} 2cq_i + \frac{1}{n}\sum_{i=1}^{n} q_i$$

which we can simplify using $\frac{\sum_{i=1}^{n} q_i}{n} = c$ to get

$$c^2 - 2c\frac{1}{n}\sum_{i=1}^{n} q_i + \frac{1}{n}\sum_{i=1}^{n} q_i = c^2 - 2c^2 + c = c - c^2,$$

which is what we wanted to show. □

Appendix E. Mathematical proof impossibility of Brier score 0

Recall the assumption.

Assumption 1. *Assume at least one of the true probabilities q_i are in $(0,1)$.*

Lemma E.1. *Let $n \in \mathbb{N}_{\geq 1}$, and let $y = (y_1,\cdots,y_n)$ be a realisation of a sequence of independent random variables, where $Y_i \sim Bern(q_i)$. If assumption 1 holds, the Brier score of the perfect prediction $p_{perf} = (q_1,\cdots,q_n)$ is bigger than zero, i.e.,*

$$BS(p_{perf}, y) > 0.$$

Proof. Denote by $q = (q_1,\cdots,q_n)$ the vector of true probabilities, which also determines the perfect prediction vector $p_{perf} = q$. Assume we reordered them such that q_1 is in $(0,1)$, which holds by assumption assumption 1. Define the following constant

$$\varepsilon := \min\{q_1, 1-q_1\}$$

By reordering and assumption, $\varepsilon > 0$. We can bound $BS(p_{perf}, y)$ from below as follows

$$0 < \frac{\varepsilon^2}{n} \leq BS(p_{perf}, y).$$

□

Appendix F. More details about the simulations

F.1. **More details for the simulation via the ADEMP framework.** We give more details on the simulations used in ADEMP framework [10]. It took roughly 45 min to run it on a Apple Mac Studio with an M2 Max chip (30-core GPU, 12-core CPU, 32 GB RAM, 512 GB SSD) running macOS 14.5. The R-code is available in the Quarto document on Github: Link

F.1.1. *Aims.*

- The main aim is to compare Brier score across different settings both for true probabilities as well as predictions, which are based on the perfect prediction with some noise or bias added through median, quantile, and violin plots.
- A secondary aim is to compare Brier score to the mean incident Brier score $\bar{y} - \bar{y}^2$, as well as to compare it to the calibration in the large (CIL).

F.1.2. *Data Generating Mechanism.*

(a) y_i entered in the Brier score

We sample true values for q_i under some distributions which are subsequently used to simulate $Y_i \sim Bern(q_i)$. The sample distribution for q_i are based on the following:

- $Unif(a,b)$, where $(a,b) \in \{(0,1),(0,0.2)\}$.
- $Beta(\alpha,\beta)$, where $(\alpha,\beta) \in \{(2,5),(5,5),(1,3)\}$.
- Osteoporosis: Logistic regression model based on NHANES 2007/2008 [6] data using the nhanesA-package [1] with complete case analysis, resulting in 298 osteoporosis entries among 4253 observations.
  - Outcome: Osteoporosis.

  - Predictors: Vitamin D, calcium, weight, height, smoking, number of persons in household, age, US citizen status, education, gender. The following were coded as restricted cubic splines using the rcs function from the rms R-package [9] with 3 knots and default knot placement, in which case they are placed at the 5th, 50th, and 95th percentile: Vitamin D, calcium, age.
  - Preprocessing and description of variables: Further coding of NHANES variables was as follows. For Osteoporosis and "Smoking/nicotine product use in the last 5 days" only "Yes" and "No" answers were coded as binary outcome, the others as NA that were removed subsequently. Vitamin D, calcium, weight, height, age, gender, US citizen status were taken as is. For education "Don't Know" was excluded but others left as is.
- Smoking: Logistic regression model based on NHANES 2007/2008 [6] data using the nhanesA-package [1] with complete case analysis, resulting in 1122 smoking entries among 4253 observations.
  - Outcome: Smoking.
  - Predictors: Vitamin D, calcium, bmi, osteoporosis, number of persons in household, age, US citizen status, education, gender. The following were coded as restricted cubic splines using the rcs function from the rms R-package [9] with 3 knots and default knot placement, in which case they are placed at the 5th, 50th, and 95th percentile: Vitamin D, calcium, bmi, age.
  - Preprocessing and description of variables: Further coding of NHANES variables was done as described above in the Osteoporosis model.

Remark F.1. Using complete-case analysis for NHANES rather than correcting for missingness does not materially affect the interpretation of the simulations. It leads to minor shifts in the distribution of true probabilities and outcomes.

(b) Predictions p_i entered in the Brier score

The predictions entered are functions of the q_i, and the following settings are considered:

- Perfect: $p_i = q_i$, i.e., perfect predictions.
- +0.1: $p_i = q_i + 0.1$, i.e., slightly biased predictions.
- $+Unif(-0.1, 0.1)$: $p_i = q_i + X_i$, where $X_i \sim Unif(-0.1, 0.1)$, i.e., disturbed but unbiased predictions.
- $+(1 - 2Bern(1/2)) \cdot 0.1$: $p_i = q_i + (1 - 2X_i) \cdot 0.1$, where $X_i \sim Bern(1/2)$, i.e., slightly disturbed but unbiased predictions.
- Logit + Normal(0,0.5): $p_i = \text{logit}^{-1}(\text{logit}(q_i) + X_i)$, $X_i \sim \mathcal{N}(0, 0.5)$, i.e., symmetric logit-scale perturbation for the predictions.

In case the p_i are smaller than zero then p_i is set to zero, and if they are bigger than one set to one.

Remark F.2. Note that truncating predictions to the interval $[0, 1]$ can introduce truncation bias, particularly when true probabilities lie near 0 or 1, and that this affects both calibration and the effective error distribution. The average fraction of trimming is given in the point below.

(c) Sample Size

Sample sizes considered are $n = \{300, 1000\}$.

(d) Number of DGM Scenarios and Simulation Runs

- $|\#$ options for dist.Y$| \cdot |\#$ options for dist. p$| = 7 \cdot 5 = 35$ scenarios.
- $N = 5000$ simulation repetitions per scenario.

F.1.3. *Estimand/Target of Analysis.*

- Distribution, median, quantiles of observed Brier score.

F.1.4. *Methods.* **(a) Basis of Simulations**

The simulation is run as a Monte-Carlo simulation in R, where for the two settings based on NHANES data, the q_i are based on the predicted value of the logistic regression for the corresponding observation, and the q_i are subsampled without replacement. For the other distributions the q_i are sampled i.i.d. from the respective distribution.

(b) Performance Measures

- distribution, median, and central 90% interval for observed Brier scores in violin plot.
- estimate the probability that $\bar{y} - \bar{y}^2 > BS_{perf}$.
- violin plot of $\bar{y} - \bar{y}^2 - BS_{perf}$.

F.2. **Average fraction of trimming.** The results for $n = 300$ and $n = 1000$ are the same. The perfect prediction and the logit perturbed predictions didn t have any trimming.

Table 1. Overview of trimmed fractions by true distributions and corresponding error distributions with nonzero entries for $n = 300, 1000$.

**True distribution**	**+Unif**$(-0.1, 0.1)$	**+0.1**	$+(1 - 2Bern(1/2)) \cdot 0.1$
$Beta(1,3)$	0.07	0.00	0.14
$Beta(2,5)$	0.02	0.00	0.06
$Beta(5,5)$	0.00	0.00	0.00
Osteoporosis	0.30	0.00	0.39
Smoking	0.03	0.00	0.08
$Unif(0, 0.2)$	0.13	0.00	0.25
$Unif(0, 1)$	0.05	0.10	0.1

References

[1] Laha Ale, Robert Gentleman, Teresa Filshtein Sonmez, Deepayan Sarkar, and Christopher Endres. nhanesa: achieving transparency and reproducibility in nhanes research. *Database*, Apr 15, 2024.
[2] Herbert Amann and Joachim Escher. *Analysis I*. Birkhauser Basel, January 2005.
[3] Peter C. Austin and Ewout W. Steyerberg. The integrated calibration index (ici) and related metrics for quantifying the calibration of logistic regression models. *Statistics in Medicine*, 38(21):4051–4065, 2019.
[4] Simon Byrne. A note on the use of empirical AUC for evaluating probabilistic forecasts. *Electronic Journal of Statistics*, 10(1):380 – 393, 2016.
[5] Frederique Chammartin, Linard Hoessly, Michael Koller, Peter Werner Schreiber, Dionysios Neofytos, Jaromil Frossard, Alexander Leichtle, and Simon Schwab. Coverage - comparing variable and feature selection strategies for prediction - protocol of a simulation study in low-dimensional transplantation data, 2025.
[6] Centers for Disease Control and Prevention (CDC). Nhanes 2007-2008, 2009.
[7] Daniel Friedman. Effective scoring rules for probabilistic forecasts. *Management Science*, 29(4):447–454, 1983.
[8] H.O. Georgii. *Stochastics: Introduction to Probability and Statistics*. De Gruyter textbook. Walter De Gruyter, 2008.
[9] Frank E Harrell Jr. *rms: Regression Modeling Strategies*, 2025. R package version 7.0-0.
[10] Tim P. Morris, Ian R. White, and Michael J. Crowther. Using simulation studies to evaluate statistical methods. *Statistics in Medicine*, 38(11):2074–2102, 2019.
[11] E.W. Steyerberg. *Clinical Prediction Models: A Practical Approach to Development, Validation, and Updating*. Statistics for Biology and Health. Springer International Publishing, 2019.
[12] Ben Van Calster, David J. McLernon, Maarten van Smeden, Laure Wynants, Ewout W. Steyerberg, Patrick Bossuyt, Gary S. Collins, Petra Macaskill, David J. McLernon, Karel G. M. Moons, Ewout W. Steyerberg, Ben Van Calster, Maarten van Smeden, Andrew J. Vickers, and On behalf of Topic Group 'Evaluating diagnostic tests and prediction models' of the STRATOS initiative. Calibration: the Achilles heel of predictive analytics. *BMC Medicine*, 17(1):230, December 2019.
[13] Tjeerd van der Ploeg, Peter C. Austin, and Ewout W. Steyerberg. Modern modelling techniques are data hungry: a simulation study for predicting dichotomous endpoints. *BMC Medical Research Methodology*, 14(1):137, December 2014.
